# An investigation of the effect of mindfulness-integrated cognitive behavior therapy on demoralization, body image, and sexual function in Iranian women with breast cancer: a randomized controlled trial

**DOI:** 10.1007/s00432-024-05655-z

**Published:** 2024-03-14

**Authors:** Mahboobeh Soleymani Moghadam, Aliakbar Parvizifard, Aliakbar Foroughi, Seyed Mojtaba Ahmadi, Negin Farshchian

**Affiliations:** 1 Department of Clinical Psychology, Kermanshah University of Medical Sciences, Kermanshah, Iran; 2 Department of Radiation Oncology, Faculty of Medicine, Kermanshah University of Medical Sciences, Kermanshah, Iran

**Keywords:** Mindfulness-integrated cognitive behavior therapy, Breast cancer, Demoralization, Body image, Sexual function

## Abstract

**Background:**

Breast cancer is an extremely unpleasant and unbearable experience that can have a profound impact on a person’s life. Compared to other types of cancer, breast cancer has a more severe psychological impact on women.

**Purpose:**

This study aimed to investigate the effect of mindfulness-integrated cognitive behavior therapy on demoralization, body image, and sexual function in Iranian women with breast cancer.

**Method:**

A sample of 30 women with breast cancer were randomly divided into intervention and control groups. The research was conducted in the oncology division of Imam Reza Hospital in Kermanshah by the clinical trial method with a two-group pretest–posttest design and a 2 month follow-up. Participants in the intervention group received Mindfulness-integrated cognitive behavior therapy for eight sessions. The intervention was carried out individually in weekly 60 min sessions. While the control group received self-help treatment (through an educational book). A demographic questionnaire, Demoralization Scale (DS-II), Body Image Scale (BIS), and Female Sexual Function Index (FSFI) were used to collect data. For data analysis, means and standard deviations were calculated and repeated measures and the Bonferroni test was conducted using SPSS 26.

**Results:**

The results showed the effectiveness of mindfulness-integrated cognitive behavior therapy on demoralization, body image, and sexual function (p < 0.05). Concerning demoralization in the intervention group, the pre-test mean was 16.73 ± 3.33, and it reduced to 11.93 ± 1.49 in the post-test. The body image mean score showed a decreasing trend in the intervention group, from 12.47 ± 1.88 in the pre-test to 8.80 ± 3.21 in the post-test. The mean score for sexual function showed an increasing trend, increasing from 18.06 ± 2.29 in the pre-test to 23.07 ± 0.91 in the post-test. There was no significant difference in the mean score of the post-test compared to the pre-test and follow-up in the control group (p < 0.05).

**Conclusion:**

MICBT can be used in conjunction with pharmaceuticals and medical treatments to improve the psychological symptoms of women with breast cancer, according to this study’s results.

*Trial registration* (IRCT20160103025817N6). 2022-04-06.

## Introduction

Breast cancer is the most common cancer among women worldwide (Shergill et al. 2022), and its incidence rate continuously increases (Liu et al. 2022). In spite of research advancements and innovative treatments, the mortality rate remains 15% (Bach et al. 2021). Breast cancer starts almost a decade earlier in Iranian women than their counterparts in developed countries (Isfahani, Arefy, and Shamsaii 2020). About 7000 new breast cancer cases are diagnosed each year in Iran (Doori et al. 2022), with the majority of patients between the ages of 40 and 49 (Alinejad Mofrad, Fernandez, Lord, and Alananzeh 2021). Patients face challenging periods during and after treatment and suffer from physical and psychological symptoms (Sakki et al. 2022). As a result of treatments such as surgery, chemotherapy, radiotherapy, or a combination of these treatments (Rani, Joseph, and Dhankhar 2023), patients may experience complications such as digestive system dysfunction, sexual dysfunction, organ dysfunction, fatigue, and pain (Liu et al. 2022). Compared to other types of cancer, breast cancer has more adverse psychological effects on women (Isfahani, Hossieni Zare, and Shamsaii 2020). Yet, demoralization is one of the psychological issues in women with breast cancer that has received less attention (Cheng et al. 2019). Demoralization is a syndrome of existential distress and hopelessness (Quintero Garzón, Hinz, Koranyi, and Mehnert-Theuerkauf 2021). People with this condition experience low morale, lack meaning and purpose in life, and feel helpless and being trapped (Murri et al. 2020). Studies have shown that 23.7–88.8% of patients with cancer experience different degrees of demoralization (W.-J. Wu et al. 2021). There is a link between demoralization and higher levels of worry, negative coping styles, and low quality of life in breast cancer patients (Cheng et al. 2019). In addition, studies have shown that women with breast cancer are subject to changes in body image and sexual function, and activity after treatment (Chang, Lin, et al. 2022). Patients with breast cancer who undergo surgery not only have to bear the trauma of cancer itself but also have to face the psychological trauma caused by the body image defect (due to the loss of the breast) (Li et al. 2022). Body image in breast cancer patients differs from other patients with different types of cancer as the outcome of treatment, including breast surgery interventions, has the greatest impact on women from a psychosocial point of view (T.-Y. Wu et al. 2019). Studies have reported 15–30% body image disturbance in women with breast cancer (Türk and Yılmaz 2018). The study also found that 100% of Iranian women who underwent mastectomy had a negative body image (Bagheri and Mazaheri 2015). Body image issues are strongly associated with sexual function in breast cancer patients (Sebri et al. 2021). The majority of patients report that breast cancer and its related treatments reduce their desire for sex and its frequency (Chang et al. 2019). Therefore, sexual dysfunction is related to both biological factors (such as hormonal changes, pain, and fatigue) and psychological factors (such as negative body image, depression, and anxiety) (Ljungman et al. 2018). Iranian women with breast cancer reported 52 and 84% sexual dysfunction before and after cancer treatment, indicating a significant decline in sexual function (Harirchi, Montazeri, Zamani Bidokhti, Mamishi, and Zendehdel 2012). However, for women, sexuality goes beyond the ability to have sex and includes body image, femininity, desirability, and childbearing abilities. It also has emotional, intellectual, and socio-cultural components (Henson 2002). In their culture, Iranian women are conservative regarding sexual issues, and expressing sexual issues is considered shameful (Momeni et al. 2023). According to Iranian cultural norms, which promote women's passiveness in sexual activities, women are prohibited from expressing their sexual problems and encouraged to prefer the sexual needs of men. As a consequence, mastectomy women are experiencing more sexual problems (Alinejad Mofrad et al. 2021). Although all cancers can affect sexual relationships and intimacy, breast cancer patients have special concerns (Henson 2002). Many interventions have been developed to improve these symptoms in cancer patients, including mindfulness-based interventions that have been shown to be promising (Hydeman, Ernhout, Attwood, and Hong 2022). Mindfulness-integrated cognitive behavior has attracted the attention of some researchers in recent years (Bahrani, Zargar, Yousefipour, and Akbari 2017). It includes a set of evidence-based techniques to increase self-awareness, self-control, and self-efficacy in various areas of life (Pouyanfard et al. 2020). MICBT employs a theoretical integration approach that incorporates the core components of learning, self-awareness, acceptance, and disengagement from the therapeutic relationship and habitual response sets. In a step-by-step process, Buddhist and Western psychology are integrated within a transtheoretical multidisciplinary framework. The practice has been found to be effective in treating physical and emotional problems and improving prosperity and well-being (Cayoun, Francis, and Shires 2018). The goal of MICBT is to help patients learn to regulate their emotions and attention and use these skills to manage their problems by combining mindfulness therapy and cognitive behavioral therapy (Pouyanfard, Mohammadpour, akbar Parvizifard, and akbar Foroughi 2019). Mindfulness skills have also been shown to support effective communication (Cayoun et al. 2018). Patients may benefit from MICBT treatment since breast cancer is known to be a relational cancer (Bigdeli Shamloo, Elahi, Asadi Zaker, Zarea, and Zareiyan 2023). Sexual dysfunction is often caused by breast cancer drug treatments due to side effects such as ovarian failure, decreased vaginal moisture, vasoconstriction, and decreased libido (Momeni et al. 2023). Also, affected patients have a negative understanding of their body image, which affects their marital relationships (Moosazadeh, Hamzehgardeshi, Elyasi, Janbabai, and Rezaei 2017). Because women are considered the core of a family in Iran as wives and mothers (Alinejad Mofrad et al. 2021), MICBT treatment intervention was provided to them. This treatment can be implemented both individually and in groups. However, the more severe the patient's symptoms, the more traumatized they are, and especially if their injury includes interpersonal or social characteristics, the more benefit they receive from individual therapy (Cayoun et al. 2018). Since breast cancer patients exhibit different characteristics and symptoms, the present study was conducted individually. Psychological symptoms of diseases such as multiple sclerosis have been used to determine the effectiveness of mindfulness-integrated cognitive behavior therapy in a group (Pouyanfard et al. 2020) (Bahrani et al. 2017). However, To the best of our knowledge, no individual study has investigated the effectiveness of MICBT on psychological symptoms in women with breast cancer. As a symbol of sex, femininity, and motherhood, mastectomy can cause demoralization, body image defects, and sexual dysfunction in affected women. Patients can benefit from MICBT treatment since it is taboo to discuss sexual issues in Iran. Therefore, this study aimed to investigate the effect of mindfulness-integrated cognitive behavior therapy on demoralization, body image, and sexual function in Iranian women with breast cancer.

## Materials and methods

### Study design

**Fig. 1:**
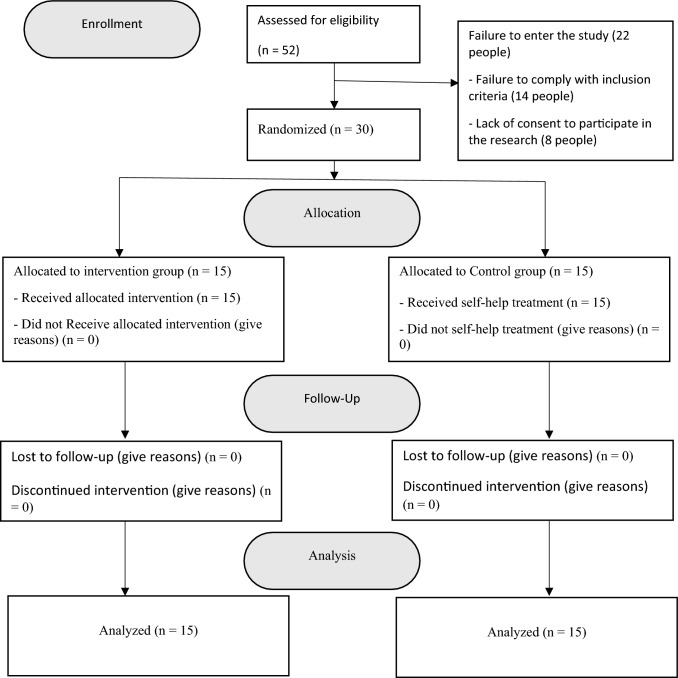
Study Flowchart

A clinical trial design with pre-tests and post-tests and a 2 month follow-up were used in the current study. The intervention group received mindfulness-integrated cognitive behavior therapy, and the control group received self-help therapy (one volume of an educational book). Data were collected with questionnaires before the intervention, immediately after the intervention and 2 months after the intervention. The research procedure is demonstrated in the flowchart in Fig. 1.

### Statistical population and sampling method

The population studied in the present study included all breast cancer patients referred to Imam Reza Hospital in Kermanshah city in 2021, whom an oncologist diagnosed. Purposive sampling was used to select the participants, and then a quadruple-type random block method was used to assign them to intervention and control groups.

### Sample size

The sample size was estimated using the following formula based on previous studies (Nabipoor Gisi, Rafieepoor, and Haji Alizadeh 2019). Alpha was considered as 0.05 and beta as 0.2. Since 15 and more participants were selected in most of the articles (Park et al. 2020), 15 participants were considered in each group in the present study.

$$\frac{\left(1-\frac{a}{2}+1+\beta \right)\left({SD}_{1}+{SD}_{2}\right)2}{\left({M}_{1}-{M}_{2}\right)2}$$

### Inclusion and exclusion criteria

The inclusion criteria were as follow: being between 30 and 60 years old, having the ability to read and write and participate in treatment sessions, receiving a breast cancer diagnosis more than 6 months ago, being married, having informed consent to participate in the research and completing the informed consent form, not suffering from other chronic physical diseases such as diabetes and thyroid disorders, not suffering from severe mental diseases including schizophrenia spectrum disorders, bipolar type 1 and severe personality disorders, not participating in psychotherapy sessions since 6 months prior the treatment, no history of other cancers, no problems with hearing and vision, no history of drug use. Also, the criteria for exclusion from the research included the following: being absent for more than two intervention sessions, metastasis in other parts of the body during the implementation of the research, unwillingness of the participants to continue the treatment process, participation in psychotherapy sessions and other support groups at the same time with the research period.

### Instruments

Demographic Information Questionnaire: This questionnaire was made by researchers and included age, educational level, duration of disease, and type of surgery (mastectomy, lumpectomy).

### Demoralization scale (DS-II)

The Demoralization Scale (DS-I) is a self-report scale that was first validated in 2004 by David Kissane in people with advanced cancers (Kissane et al. 2004). It was then re-evaluated by Robinson et al. (2016). DS-II consists of 16 questions rated on a 3-point Likert scale, including 0 (never), 1 (sometimes), and 2 (often). Higher scores indicate greater demoralization (0–32). The authors of the original DS-II scale defined the following cutoff criteria for the total score: low demoralization (0–3), moderate demoralization (4–10), and high demoralization (equal to or greater than 11) (Koranyi et al. 2021). This scale includes two 8-item subscales. The meaning and purpose subscale includes questions (1–2–3–5–6–7–13–14), and the confusion and coping ability subscale includes questions (4–8–9–10–11–12–15–16) (Robinson et al. 2016). The demoralization questionnaire was standardized by the first author and colleagues after administering the test to 240 women with breast cancer, and Cronbach’s alpha coefficient was 0.81, which indicates the good reliability of this scale.

### Body image scale (BIS)

This valid questionnaire consists of 10 questions and measures concerns about body image, including emotional, behavioral, and cognitive issues. It is widely used in cancer fields and especially in breast cancer research. Participants rate their agreement on a four-point Likert scale (0 = not at all, 3 = very much) with statements such as (Do you feel self-conscious about your appearance?). Total scores range from 0 (no concern) to 30 (high concern about body image) (Todorov, Sherman, Kilby, and Australia 2019). It was localized in Iran by Rajabi et al. (2015), and they reported Cronbach’s alpha coefficient of 0.70 for this instrument (Rajabi, Kaveh Farsani, Fadaei Dehcheshmeh, and Jelodari 2015).

### Female sexual function index (FSFI)

The Female Sexual Functioning Index (FSFI) is a 19-item scale scored on a five-point Likert-type scale. It includes six separate areas of female sexual function, namely desire, arousal, lubrication, orgasm, satisfaction, and sexual pain. Scores range from 0 to 36. Higher scores indicate better sexual function (Chang, Lin, et al. 2022). This tool was localized in Iran by Fakhri et al., and has demonstrated to be a reliable and valid instrument with good psychometric properties (Fakhri et al. 2012).

#### Method

The research was conducted in the oncology division of Imam Reza Hospital in Kermanshah by the clinical trial method with a two-group pretest–posttest design and a 2 month follow-up. A total of 30 women with breast cancer were recruited by purposive sampling, then 15 were assigned to an intervention group and 15 to a control group by quadruple-type random blocks. Quadruple blocks of intervention (A) and control (B) groups were created (AABB, ABAB, ABBA, BBAA, BABA, BAAB). Subsequently, numbers 1–6 were assigned to cards, which were then placed into envelopes. A random selection of cards was made, forming a random string based on the chosen sequence. Participants from the intervention and control groups were assigned to the study according to this sequence. Blinding was not used in the present study. Participants in the intervention group received mindfulness-integrated cognitive behavior therapy, and participants in the control group received self-help therapy (one volume of an educational book). The intended intervention was based on the book written by Cayoun (Cayoun et al. 2018) titled the clinical handbook of Mindfulness-integrated Cognitive Behavior Therapy: a step-by-step Guide for Therapists). The study was conducted as an individual intervention in 8 sessions of 60 min by the first author. Table 1 provides more details of the contents of the therapy sessions.

**Table 1:** The curriculum for MICBT sessions

Sessions	Content
First	Introduction about meetings and rules, an overview of MICBT, the concept and principles of mindfulness and mindful breathing
Second	Mindful breathing (continued), overview of several concepts of MICBT, (such as situation, sensory perception, evaluation, emotions and body reaction), internal causes of disturbing thoughts and their correction, part-by-part body scanning
Third	Part-by-part body scanning (continued), explanation about body sensations, informal practice
Forth	Body-scanning exercises (cont.), introduction of some CBT components in MICBT (such as exposure techniques), coping with unpleasant emotions using the Subjective Units of Distress Scale (SUDS) (a form used to measure exposure to target events) through Bipolar exposure (imaginary exposure to unpleasant situations)
Fifth	Body scanning exercises (continued), SUDS check
Sixth	Body scanning exercises (continued), interpersonal skills, assertiveness and role playing
Seventh	Introducing the concepts of compassion and empathy, loving-kindness meditation
Eighth	Review and assessment

### Data analysis

The obtained data were analyzed using SPSS 26 software. The chi-square test was used to check the homogeneity of education, and Fisher’s exact test was used to check the homogeneity of the type of surgery. Background variables of age and duration of infection, were investigated with descriptive indices, and the homogeneity of the groups in terms of age and duration of the disease was investigated with the independent samples t-test. The results were analyzed using repeated measures ANOVA. The scores in the pre-test were compared to those in the post-test and follow-up using Bonferroni’s post-hoc test. The maximum alpha error level to test the hypotheses was considered to be 0.05 (p ≥ 0.05).

### Ethical considerations

Before starting the study, a meeting was held to explain the project to the patients. In this meeting, ethical issues were presented, and research was explained to the patients. Then, all participants completed informed consent forms, and participants were assured that their results and identity would be kept confidential and that under no circumstances would their health information be shared with anyone except the medical staff. In this study, all health protocols related to Covid-19 were observed. Also, self-help training (receiving a training book) was used for the control group in order to comply with ethical principles. This study was approved by the Research Ethics Committee of Kermanshah University of Medical Sciences (ethical code: IR.KUMS.MED.REC.1400.088). This study has also been registered in the Iranian Registry of Clinical Trials (code: IRCT20160103025817N6).

#### Results

The sample size of each group is 15 people. The significance level of the chi-square test for education and Fisher’s exact test for the type of surgery showed that there was no significant difference between the background characteristics of the two groups (p < 0.05). Background variables of age and duration of infection, were investigated with descriptive indices, and the homogeneity of the groups in terms of age and duration of the disease was investigated with the independent samples t-test. The results showed that the average age in the intervention group was equal to 42.53, and in the control group was equal to 44.07. Therefore, the difference between the means was not significant and indicated the homogeneity of age in the groups (p < 0.05). The mean score for the disease duration in the intervention group was equal to 11.20, and in the control group, it was equal to 11, which indicated the equality of the two groups in terms of this underlying variable (p < 0.05) (Table 2).

**Table 2:** The demographic characteristics of patients in intervention and control groups^a^

Variables	Conditions	Groups		Homogeneity test		Independent t-test	
		Intervention group	Control group	Statistic	P value	Statistic	P value
Educational level	Junior high school diploma	2 (13.3)	4 (26.7)	1.56^b^	0.458		
	High School Diploma	7 (46.7)	4 (26.7)				
	Undergraduate	6 (40)	7 (46.7)				
Type of surgery	Mastectomy	11 (73.3)	12 (80)	0.186^c^	0.666		
	Lumpectomy	4 (26.7)	3 (20)				
Age		8.24 ± 42.53	7.95 ± 44.07			0.52	0.608
Duration of the disease		1.78 ± 11.20	1.41 ± 11.00			0.34	0.736

^a^Values are expressed as No (%) or mean ± Standard deviation

^b^Statistic in Chi-square test

^c^Statistic in Fisher’s exact test

The demoralization score showed a decreasing trend in the intervention group, Also, the body image scores showed a decreasing trend in the intervention group. The mean scores of sexual function showed an increasing trend. According to the independent group’s T-test, all three variables had similar mean scores during the pre-test and were almost equal in both groups. Accordingly, the pre-test scores of the variables were homogeneous in the two groups (p < 0.05). MICBT significantly reduced demoralization and negative body image in the intervention group compared to the control group. Additionally, MICBT significantly improved sexual function among intervention group participants compared to those in the control group. Based on the findings, the effect of time and group and the interaction effect on the mean of demoralization, body image, and sexual function were statistically confirmed (p < 0.05). It was found that the group had a significant effect, and the mean score of demoralization and body image of the intervention group decreased significantly compared to the control group. In contrast, the mean of sexual function increased significantly, indicating the effectiveness of Mindfulness-integrated cognitive behavior therapy on all three variables (p < 0.05). Also, the effect of time was significant, which means a significant change in the mean score of all three variables in pre-test, post-test, and follow-up (p < 0.05). The significance of the interaction effect showed the difference in scores of all three variables in both groups (Table 3).

**Table 3:** Independent t-test, ANOVA, Mean and standard deviation of Patients in Intervention and Control groups

Variables	Conditions	Groups		Independent t-test		ANOVA		
		Intervention^a^	Control^a^	Statistic	P value	F value	P value	Effect size
Demoralization	Pre-test	16.73 ± 3.33	17.27 ± 3.22	0.45	0.659	10.92	0.003	0.281
	Post-test	11.93 ± 1.49	16.87 ± 3.31	5.26	> 0.001^***^	24.74	> 0.001	0.469
	Follow-up	12.13 ± 2.36	16.20 ± 3.59	3.67	0.001^***^	9.62	0.004	0.256
Body image	Pre-test	12.47 ± 1.88	10.60 ± 4.29	1.54	0.134	5.153	0.021	0.163
	Post-test	8.80 ± 3.21	10.47 ± 4.39	1.19	0.245	22.25	> 0.001	0.443
	Follow-up	9.27 ± 3.24	11 ± 4.29	1.25	0.222	36.78	> 0.001	0.568
Sexual function	Pre-test	18.06 ± 2.29	18.43 ± 2.43	0.42	0.674	29.26	> 0.001	0.511
	Post-test	23.07 ± 0.91	18.61 ± 2.35	6.86	> 0.001^***^	36.85	> 0.001	0.568
	Follow-up	23.41 ± 0.93	17.62 ± 2.04	10.01	> 0.001^***^	67.64	> 0.001	0.707

^***^p =  ≤ 0/001

^a^Values are expressed as mean ± Standard deviation

The test result Bonferroni's post hoc test showed that the mean score of demoralization and body image was significantly lower in the post-test and follow-up than the pre-test in the intervention group (p < 0.05). Also, the result of the Bonferroni test showed that the mean score of sexual function during the post-test and follow-up was significantly higher than the pre-test in the intervention group (p < 0.05). There was no significant difference in the mean score of the post-test and follow-up compared to the pre-test in the control group (p < 0.05) (Table 4).

**Table 4:** Bonferroni’s test

Variable	Comparison	Intervention group			Control group		
		Mean difference	Standard error	P value	Mean difference	Standard error	P value
Demoralization	Pre-test with post-test	4.80	0.656		0.40	0.190	0.162
	Pre-test with follow-up	4.60	1.06	0.002	1.07	0.408	0.061
	Post-test with follow-up	− 0.20	0.656	1	0.67	0.303	0.136
Body image	Pre-test with post-test	3.67	0.494	> 0.001	0.13	0.091	0.493
	Pre-test with follow-up	3.20	0.527	> 0.001	− 0.40	0.273	0.493
	Post-test with follow-up	0.47-	0.215	0.144	− 0.53	0.236	0.122
Sexual function	Pre-test with post-test	− 5.01	0.651	> 0.001	− 0.18	0.180	1
	Pre-test with follow-up	− 5.35	0.613	> 0.001	0.81	0.430	0.246
	Post-test with follow-up	− 0.34	0.134	0.070	0.99	0.357	0.046

## Discussion

This study aimed to investigate the effectiveness of mindfulness-integrated cognitive behavior therapy in demoralization, body image, and sexual function in women with breast cancer. The results of the present study showed that mindfulness-integrated cognitive behavior therapy has favorable effects on the psychological symptoms of breast cancer patients and reduces the symptoms of demoralization and body image in patients. The results of the present study are consistent with previous studies, including Chang et al.’s study (Chang, Chiu, et al. 2022), Pintado et al.’s study (Pintado and Andrade 2017), Rasouli et al.’s study (Rasouli et al. 2023), Sharbaf et al.’s study Showing Mindfulness can improve body image in breast cancer survivors. Mindfulness is defined as the awareness of the body. This awareness is an interactive and dynamic process that includes understanding bodily states, actions, and feelings (Pintado and Andrade 2017). It has been found that patients with higher levels of mindfulness can more quickly accept adverse physical and mental problems during treatment and form rational thinking patterns through self-awareness to relieve negative emotions (YuYu, Shan, and JingJun 2023). As a result of paying attention to the present moment and accepting it without judgment, mindfulness enhances body awareness and reduces rumination about body image (Pintado and Andrade 2017). In fact, people skilled at recognizing basic body sensations and accepting them as they happen are more skilled at regulating emotions that usually manifest themselves in the form of thoughts and feelings (Bahrani et al. 2017). Hence, mindfulness helps people deal with emotions peacefully (Zhang et al. 2022). One of the factors that may lead to demoralization in the patient is the feeling of being a burden to others or losing independence (Belar et al. 2019). Therefore, teaching self-expression and role-playing skills, together with mindfulness exercises as coping tools, can increase patients’ ability to act, help them build self-confidence, and prepare them for future action, which in turn can increase their hope (Pouyanfard et al. 2020). Also, when the patients can observe their inner feelings, they can promote conscious action, consciously regulate bad feelings, and have better psychological management of symptoms to reduce the occurrence of demoralization syndrome (YuYu et al. 2023). Also, the present study showed that mindfulness-integrated cognitive behavior therapy improved sexual performance in patients. However, previous studies including Chang et al.’s study Showing that mindfulness-based stress reduction could improve parts of in patients with breast cancer sexual function i.e. arousal, lubrication and satisfaction (Chang, Lin, et al. 2022). Also, Bagherzadeh et al.’s study Showing that mindfulness intervention can affect aspects of sexual performance with a psychological origin and may not affect all aspects (Bagherzadeh et al. 2021). Since breasts symbolize femininity and sexuality, procedures like lumpectomy and mastectomy affect the sexual life of women with breast cancer and their husbands (Jeng et al. 2020). Sexual relationship contributes significantly to marital satisfaction. And its Dissatisfaction can result in negative consequences including feelings of depression and despair, and may even lead to marital disruption (Shandiz et al. 2016). Mindfulness can increase direct access to bodily sensations by training attention and reducing negative self-evaluation. Moreover, it can reduce anxiety, guilt, self-criticism, and hopelessness, which may prevent women from becoming sexually aroused (Brotto and Basson 2014). As a result, a lifestyle based on mindfulness strengthens collaborative relationships and limits destructive behaviors based on criticism and judgment (Iannopollo et al. 2022). Also, when women practice non-judgment in sex relations, this can reflect less self-judgment and more acceptance of their partner (Brotto and Basson 2014). Therefore, patients with a high level of mindfulness can treat their illness and life with a peaceful and accepting attitude. Also, mindfulness helps patients to improve their emotional stability and ability to cope with stress to achieve good psychological adjustment and personal growth (YuYu et al. 2023). Despite this, we believe that husbands and children's support played an important role in improving the situation because most Iranian women are Muslim, and mothers are at the center of families. Furthermore, their religious beliefs helped them endure pain and cope with psychological problems, such as believing that their illness was the will of God. As a result, MICBT can be used in clinical practice by specialists in the field of breast cancer to reduce the symptoms caused by pharmaceuticals and medical treatments. The current research had some limitations, including that some patients did not want to cooperate due to inappropriate psychological conditions and fatigue caused by chemotherapy. Therefore, a meeting plan was implemented based on the patients’ available times to get their cooperation. Another limitation is related to the fact that the type of drug treatment used to treat the patients (chemotherapy, radiation therapy, hormone therapy) was different. This difference in the drug treatment procedure may lead to different results for the patients. Furthermore, this research is limited by the exclusion of important background variables such as job, history of hospitalization, family history, and the intensity of the emotional relationship with the husband. And, the last limitation was using a heterogeneous sample (mastectomy or lumpectomy) due to the unavailability of a homogeneous sample. Since breast cancer is known as a relational disease and affects marital relations, it is recommended that their husbands also participate in this treatment at the same time. As a result, they gain a better understanding of their wives' emotional and physical needs, become more adaptable to changes in their spouses, have better sexual relations and provide better support to their wives.

## Conclusion

Breast cancer is a stressful disease that affects the psychological states of patients and worsens the health status and quality of life of affected women. In this sense, mindfulness helps to recognize, accept and improve body-related thoughts and feelings in these patients. Therefore, MICBT can be used as a complementary treatment for these patients.
